# Prevalence of aldosterone breakthrough in dogs receiving renin‐angiotensin system inhibitors for proteinuric chronic kidney disease

**DOI:** 10.1111/jvim.16573

**Published:** 2022-11-09

**Authors:** Marisa K. Ames, Shelly L. Vaden, Clarke E. Atkins, Jean‐Sebastien Palerme, Catherine E. Langston, Gregory F. Grauer, Sarah Shropshire, Christina Bove, Tracy Webb

**Affiliations:** ^1^ Department of Clinical Sciences Colorado State University College of Veterinary Medicine and Biomedical Sciences Fort Collins Colorado USA; ^2^ Department of Clinical Sciences North Carolina State University College of Veterinary Medicine Raleigh North Carolina USA; ^3^ Department of Veterinary Clinical Sciences Iowa State University College of Veterinary Medicine Ames Iowa USA; ^4^ Department of Veterinary Clinical Sciences The Ohio State University College of Veterinary Medicine Columbus Ohio USA; ^5^ Department of Clinical Sciences Kansas State University College of Veterinary Medicine Manhattan Kansas USA; ^6^ Present address: Department of Medicine and Epidemiology, School of Veterinary Medicine University of California, Davis Davis California USA; ^7^ Present address: Four Seasons Veterinary Specialists Loveland Colorado USA

**Keywords:** mineralocorticoid receptor antagonists, nephrology, renin‐angiotensin‐aldosterone system, urine aldosterone to creatinine ratio

## Abstract

**Background:**

The influence of aldosterone breakthrough (ABT) on proteinuria reduction during renin‐angiotensin system (RAS) inhibition for spontaneous proteinuric chronic kidney disease (CKD_P_) has not been determined in dogs.

**Objectives:**

Determine whether ABT occurs in dogs with CKD_P_ and if it is associated with decreased efficacy in proteinuria reduction during RAS inhibitor treatment.

**Animals:**

Fifty‐six client‐owned dogs with CKD_P_ and 31 healthy client‐owned dogs.

**Methods:**

Prospective, multicenter, open‐label clinical trial. Dogs were treated with an angiotensin‐converting enzyme inhibitor or angiotensin receptor blocker alone or in combination at the attending clinician's discretion and evaluated at 5 time points over 6 months. Healthy dogs were used to determine the urine aldosterone‐to‐creatinine ratio cutoff that defined ABT. The relationship of ABT (present at ≥50% of visits) and proteinuria outcome (≥50% reduction in urine protein‐to‐creatinine ratio from baseline at ≥50% of subsequent visits) was evaluated. Mixed effects logistic regression was used to evaluate the relationship between clinical variables and outcomes (either successful proteinuria reduction or ABT).

**Results:**

Thirty‐six percent (20/56) of dogs had successful proteinuria reduction. Between 34% and 59% of dogs had ABT, depending on the definition used. Aldosterone breakthrough was not associated with proteinuria outcome. Longer duration in the study was associated with greater likelihood of successful proteinuria reduction (*P* = .002; odds ratio, 1.6; 95% confidence interval [CI], 1.2‐2.2).

**Conclusions and Clinical Importance:**

Aldosterone breakthrough was common in dogs receiving RAS inhibitors for CKD_p_ but was not associated with proteinuria outcome.

**Abbreviations**
ABT: aldosterone breakthrough
ACEi: angiotensin converting enzyme inhibitor
ARB: angiotensin II receptor blocker
CI: confidence interval
CKD_P_
: proteinuric chronic kidney disease
IQR: interquartile range
MRA: mineralocorticoid receptor antagonist
RAAS: renin angiotensin aldosterone system
RAS: renin angiotensin system
RASi: renin angiotensin system inhibitor
SBP: systolic blood pressure
UAldo : C: urine aldosterone to creatinine ratio
UP : C: urine protein to creatinine ratio
USG: urine specific gravity
WCI/WRI: width of the confidence interval to width of the reference interval ratio

## 1 INTRODUCTION

Proteinuria is a modifiable risk factor in animals and humans with chronic kidney disease.^1^, ^2^, ^3^, ^4^, ^5^ Inhibition of the renin‐angiotensin aldosterone system (RAAS) with angiotensin converting enzyme inhibitors (ACEi) and angiotensin II receptor blockers (ARB) decreases proteinuria in dogs with spontaneous proteinuric chronic kidney disease (CKD_P_).^6^, ^7^, ^8^ Because aldosterone secretion is increased in response to renin‐angiotensin system (RAS) activation and mediates glomerular podocyte injury, it is an additional therapeutic target.^9^, ^10^ Angiotensin II is a major secretagogue of aldosterone and ACEi and ARB treatment should decrease its formation. However, in 1 study approximately 1/3 of dogs receiving an ACEi for myxomatous mitral valve disease had aldosterone concentrations that exceeded a normal population‐derived cutoff.^11^ It is not clear if this failure of RAAS inhibitors (RASi) to suppress aldosterone (aldosterone breakthrough; ABT) occurs in dogs with CKD_P_. The addition of a mineralocorticoid receptor antagonist (MRA) to an ACEi or ARB has been shown to augment proteinuria reduction in people with systemic hypertension, diabetic nephropathy, and chronic kidney disease.^12^, ^13^, ^14^ One rationale behind adding an MRA to an ACEi or ARB is to antagonize the mineralocorticoid receptor, mitigating the adverse effects of excess aldosterone. The benefit of adding spironolactone to an ACEi in dogs with heart failure caused by myxomatous mitral valve disease recently was supported by the positive results of the BEnazepril Spironolactone STudy (BESST) trial.^15^ This strategy has not been evaluated in dogs with CKD_P_.

Previous studies investigating ABT in veterinary medicine have used the urine aldosterone‐to‐creatinine ratio (UAldo : C) and a high performance liquid chromatography‐mass spectrometry‐derived RAS Fingerprint with concurrent aldosterone quantification.^11^, ^16^ Reference intervals for spot blood aldosterone concentrations in dogs are wide, likely reflecting variations associated with type of assay and patient characteristics such as hydration status, dietary sodium, and level of excitement.^17^, ^18^, ^19^, ^20^ Conversely, the urine aldosterone concentration represents hours of aldosterone secretion. A recent study showed that UAldo : C ratios determined from 24‐hour urine samples were significantly correlated with the UAldo : C determined from spot urine samples and mirrored the significant increases in serum angiotensin peptides and aldosterone concentrations during diuretic administration.^16^ The UAldo : C is therefore a useful and simple indicator of RAAS activation.

Our primary objective was to determine the prevalence of ABT in dogs with spontaneous CKD_P_ treated with an ACEi or ARB alone, or in combination. A second objective was to determine if ABT influenced proteinuria reduction during RAAS inhibition. We also measured the UAldo : C in 31 apparently healthy dogs to determine a UAldo : C cutoff for the radioimmunoassay used in the study.

## 2 MATERIALS AND METHODS

### 2.1 Study design

This study was a prospective, multicenter, open‐label clinical trial.

### 2.2 Healthy dogs

Thirty‐one apparently healthy, male and female, adult dogs were recruited at the Colorado State University Veterinary Teaching Hospital between 2016 and March 2017. Dogs could be receiving nonsteroidal anti‐inflammatory drugs, gabapentin, tramadol, fish oils, glucosamine chondroitin, intermittent antihistamines, and parasite preventatives. Administration of any other drugs led to exclusion. Additional exclusion criteria included a history of polyuria and polydipsia, cough, or known preexisting or clinicopathologic evidence of systemic disease (cardiac, endocrine, respiratory, lower urinary tract, or renal disease). Trace proteinuria was allowed if the urine specific gravity (USG) was >1.030.

### 2.3 Proteinuric dogs

Dogs presented to the internal medicine departments at 4 colleges of veterinary medicine (North Carolina State University, Iowa State University, The Ohio State University, and Colorado State University) were prospectively enrolled between 2014 and 2020. Written client consent was obtained at enrollment. The study was approved by the Animal Care and Use Committee or Clinical Trial Review Board at each institution. Dogs with CKD_P_, defined as a urine protein‐to‐creatinine ratio (UP : C) >1.0 based on the average of 2 UP : C determinations and 2 concurrent urinalyses, with inactive urine sediment and negative urine culture, were prospectively enrolled. Dogs already receiving an ACEi or ARB or both for CKD_P_ were eligible to be enrolled. Administration of an ACEi or ARB or both was initiated in dogs not already receiving anti‐proteinuric drugs. The choice of drug and dosage were at the attending clinician's discretion. Dosages could be titrated by the attending clinician at each visit, including the initial visit, in dogs already receiving a RASi. Dogs with a previous or new diagnosis of systemic hypertension (persistent systolic blood pressure [SBP] > 180 mm Hg) were included, and amlodipine was allowed. Nutritional supplements and diet were not controlled. Treatment for thromboprophylaxis, osteoarthritis, urinary incontinence, appetite stimulation, gastroprotection and parasite prevention was allowed.

Dogs were excluded if they were being treated for any disease known to cause proteinuria (hyperadrenocorticism, tick‐borne disease, heartworm disease, or neoplasia), had a positive urine culture, or were not expected to survive the 6‐month evaluation period. Prohibited medications included spironolactone, desoxycorticosterone pivalate, and diuretics.

### 2.4 Scheduled visits

#### 2.4.1 Healthy dogs

Dogs were screened using history, physical examination, SBP measurement, PCV and total solids concentration, serum biochemistry, and urinalysis. Dogs were gently restrained in right or left lateral recumbency, a cuff with a width of 30% to 40% of the distal forelimb radius was placed on the forelimb, and at least 3 measurements within 10% of each other were averaged using a Doppler blood pressure device. Owners collected urine at home on 2 consecutive days from the dog's first morning urination, before eating. If dogs were fed ad libitum throughout the day and night, this routine was not changed. Water was not restricted. Urine was collected in a sterile sample cup and then refrigerated for <72 hours before processing and storage. Samples from each dog included day 1, day 2, and mixed (equal aliquots from each day) samples. Urine from day 1 was submitted for complete urinalysis (Colorado State University Clinical Pathology Laboratory) and a separate aliquot was centrifuged, and the supernatant stored at −80°C until analysis.

#### 2.4.2 Proteinuric dogs

Dogs were evaluated at a baseline visit and again at months 1, 2, 4, and 6. At all visits, a physical examination, SBP measurement, CBC, serum biochemistry, urinalysis, UP : C, and UAldo : C were performed. Whether a Doppler or oscillometric blood pressure device was used was based on the protocol used at the participating internal medicine departments. Urine was collected by cystocentesis for all urine cultures and by cystocentesis or voiding for all other tests. Urine culture was only performed at the baseline visit.

### 2.5 Urine aldosterone and creatinine quantification

Two to 5 mL of urine were centrifuged and transferred to a −80°C freezer within 8 hours of collection and stored for future analysis. A radioimmunoassay was used to measure urine aldosterone concentration following the manufacturer's directions (Aldosterone Active Radioimmunoassay, Beckman Coulter, Inc, Brea, California) at a veterinary diagnostic laboratory (Michigan State University Veterinary Diagnostic Laboratory, Lansing, MI). This assay measures free aldosterone and the aldosterone 18β‐G metabolite (after acid hydrolysis to aldosterone). Urine creatinine concentration was determined using a standard colorimetric assay at the same laboratory.

### 2.6 Proteinuria

Confirmation of proteinuria was based on a UP : C measured from 2 separate urine samples. A single urine sample was collected at all subsequent visits to reevaluate the UP : C. Successful proteinuria reduction was defined as ≥50% decrease in the UP : C as compared to the baseline value at ≥50% of subsequent visits.

### 2.7 Definition of aldosterone breakthrough

Cutoff and baseline definitions of ABT were used.^11^, ^21^ Aldosterone breakthrough was defined as a UAldo : C >1.7 μg/g on ≥50% of visits, while receiving RASi. This cutoff was determined using the mean plus 2 SD UAldo : C (using a mixed, day 1 and day 2 urine sample) in the 31 healthy dogs. The cutoff definition could be applied to all dogs. A second baseline definition was applied to dogs that were not receiving a RASi at the initial study visit and was defined as a UAldo : C that exceeded the pretreatment baseline value on ≥50% of subsequent study visits.

### 2.8 Statistical analysis

#### 2.8.1 Healthy dogs

A double‐sided 95% reference interval (2.5% and 97.5% limits) for the UAldo : C was determined using a commercial software program (MedCalc Software, Ostend, Belgium). Data were log transformed before analysis. The 90% confidence intervals (CI) of the reference interval limits were determined using the robust method. The precision of the upper and lower limits was estimated by the ratio of the width of the 90% CI of the limits to the width of the reference interval (WCI/WRI). The Reed method was used to identify outliers. Spearman correlation was calculated for diet sodium content (percent on a dry matter basis) and the day 1 UAldo : C. The agreement for the UAldo : C from day 1 vs day 2 was evaluated using the Bland Altman 95% limits of agreement and a paired *t* test to assess differences between the 2 days. The Shapiro‐Wilk test was used to evaluate the normality of the differences. A Pearson correlation also was calculated for the paired samples from the 2 days.

#### 2.8.2 Proteinuric dogs

Dogs with ≥3 study visits were included in the analysis. Data were tested for normality using the D'Agostino and Pearson test. Descriptive statistics are presented as mean ± SD or median (interquartile range [IQR]) depending on distribution. The prevalence of ABT was determined by the number of dogs fitting a definition of ABT divided by the number of dogs in the study population. The association between ABT at ≥50% of visits (by either definition) and whether patients had ≥50% reduction in proteinuria at ≥50% of visits was evaluated using a Fisher's Exact test. The correlation between the UAldo : C and UP : C was evaluated using a Pearson correlation. The mean UP : C and change in UP : C from the first to final visit was compared among dogs receiving ACEi, ARB, those that transitioned from an ACEi to an ARB, and dogs that received both an ACEi and ARB using a the Kruskal‐Wallis test. Mixed effects logistic regression was used to model binary outcome variables, where the odds of the outcome (successful proteinuria reduction, either ≥50% reduction in UP : C at ≥50% of visits or presence of ABT) were modeled as a linear combination of the predictor variables. Binary (yes/no) variables included age (>8 years), serum creatinine concentration (>2.8 mg/dL), SBP (>160 mm Hg), and serum albumin concentration (<3 g/dL), RASi dose escalation, and RASi change from ACEi to ARB. Additional binary variables included RASi type (ACEi vs ARB) and RASi dosage (lower vs higher; lower defined as enalapril <0.8 mg/kg/day, benazepril <0.4 mg/kg/day, or telmisartan <0.75 mg/kg/day). Two models were created using either the cutoff or the baseline definition of ABT. Variables were evaluated using Akaike Information Criteria^22^ and removed if they did not pass this selection process. Significance was determined by a *P* value ≤.05. These analyses were performed using commercially available software programs (GraphPad Prism version 9.1, La Jolla, California, and R version 4.0.2, Vienna, Austria).

## 3 RESULTS

### 3.1 Urine aldosterone‐to‐creatinine ratio in healthy dogs

Thirty‐five dogs were evaluated with 4 dogs being excluded because of the diagnoses of biliary mucocele, urinary tract infection, proteinuria (other than trace with USG >1.030), or moderate subaortic stenosis. Pertinent demographic and clinical data are presented in Supporting Information. The median (IQR) UAldo : C ratios for day 1, day 2, and mixed samples were 0.64 (0.54‐0.83); 0.70 (0.47‐0.93); and 0.66 (0.46‐0.84) μg/g, respectively (Table S2). Two dogs (a 6.5‐year‐old neutered male and 8‐year‐old spayed female) had high UAldo : C ratios on both days (Figure S1). The female dog had the lowest USG recorded in the study (1.017) and the male dog had mildly increased alkaline phosphatase activity. Otherwise, clinicopathologic and physical examination findings were normal. Other than these 2 dogs, no dog had UAldo : C ratio >1.3 on any day. No outliers were detected. The UAldo : C reference interval derived from the day 1 samples was 0.23‐1.82 μg/g with 90% CI of 0.17‐0.32 and 1.27‐2.5, respectively (Table S2). The WCI/WRI of the lower and upper limits were 0.09 and 0.77, respectively.

The day‐to‐day intra‐individual coefficient of variation for the UAldo : C ratio was 36.5%, and the inter‐individual coefficient of variation was 69.0%. The difference in means (estimated bias) between UAldo : C ratios from day 1 and 2 was 0.002 with a SD of 0.29. Lower and upper limits of agreement were −0.56, and +0.57, respectively. The Pearson's correlation for day 1 and day 2 was 0.85. The Shapiro‐Wilk test supported normality of the differences (*P* = .14). A paired *t* test for day 1 and day 2 samples did not show a difference between the 2 days (*P* = .97). The mean (SD) and median (range) percentage change between the 2 days for all 31 dogs was 28% (26%) and 21% (1%‐127%), respectively. All but 3 dogs had day‐to‐day percentage changes <50%. Of the 3 dogs with day‐to‐day changes >50%, none exceeded the upper limit of the UAldo : C reference interval on either day.

### 3.2 Proteinuric dogs

Seventy‐one dogs with proteinuria were enrolled. Fifteen dogs were excluded for various reasons, including an average UP : C <1.0 on their first study visit (5 dogs), administration of a loop diuretic (1 dog), administration of mycophenolate for presumed immune‐mediated glomerulonephritis (4 dogs), death before completing at least 3 study visits (2 dogs), inadequate urine samples for ≥3 visits (2 dogs), and owner removal after the second visit (1 dog). Fifty‐six dogs had ≥3 visits and were included in the analysis. Thirty‐two dogs completed all 5 visits, 16 completed 4 visits, and 8 completed 3 visits. Because some dogs missed a mid‐point visit (i.e., did not return at the scheduled time or had an inadequate volume of urine sample), the numbers of dogs vary at each timepoint. The number of dogs evaluated at months 0, 1, 2, 4, and 6 were 56, 50, 51, 46, 45, respectively. Five of the 56 dogs died during the study. The reason for death in these dogs was euthanasia because of gastrointestinal disease in 3 dogs, euthanasia because of renal failure in 1 dog, and death at home after acute respiratory distress in 1 dog known to have myxomatous mitral valve disease.

The mean (SD) age of the dogs was 9.3 (3.0) years. There were 34 spayed females and 22 neutered males. There were 11 mixed breed dogs, 5 Yorkshire terriers, 5 Poodles (4 miniature and 1 standard), 4 Golden Retrievers, 3 each of Shetland sheepdog, Pomeranian, Boston terrier, and Cocker spaniel, 2 Boxers, and 1 each of the following: Welsh Corgi, Bloodhound, Papillion, Wirehaired Fox terrier, Whippet, Wheaton terrier, Beagle, Greyhound, Doberman, Bichon Frise, Australian cattle dog, Pit Bull terrier, Labrador retriever, Chesapeake Bay retriever, Cairn terrier, miniature Schnauzer, and French bulldog.

In addition to an ACEi or telmisartan or both, dogs received clopidogrel (n = 28), amlodipine (n = 18), aluminium hydroxide (n = 6), doxycycline (n = 3), levothyroxine (n = 3), PO cyclosporine for chronic atopy (n = 1), ophthalmic cyclosporine (n = 1), prednisone for chronic cough (n = 1), phenylpropanolamine (n = 2), probiotic (Azodyl, n = 2), rivaroxaban (n = 2), atenolol (n = 1), sotalol (n = 1), and estriol (n = 1).

### 3.3 Renin‐angiotensin system inhibitor treatment

Thirty‐four of 56 dogs (61%) already were receiving a RASi before their inclusion in the study. The median (IQR) duration of treatment was 2 (1–5) months. Twenty‐three of these dogs were receiving an ACEi, 10 were receiving telmisartan, and 1 was receiving both an ACEi and telmisartan. Twenty‐two dogs were naïve to RAS inhibition and were started on either an ACEi or telmisartan at the discretion of the attending clinician at their first visit. One dog naïve to RASi had a >30% increase in serum creatinine concentration 1 month after starting a RASi (benazepril). Fifteen dogs had a >30% increase in serum creatinine concentration between ≥1 study visits. Of these 15 dogs, 3 were receiving an ACEi, 8 were receiving telmisartan, and 4 transitioned from an ACEi to telmisartan. For the 4 dogs that transitioned to telmisartan, the increase in serum creatinine concentration was associated with this transition in 2 dogs.

Throughout the study, 28 of 56 dogs received only an ACEi (either enalapril or benazepril), 16 of 56 dogs received only telmisartan, and 12 of 56 dogs received an ACEi and telmisartan, consecutively (9 dogs transitioned from an ACEi to telmisartan during the study) or in combination (3 dogs received an ACEi and telmisartan concurrently for at least 1 study visit; Table 1). Twenty‐two dogs had at least 1, and 4 dogs had >1 dosage increase in their RASi during the study (Table 1). Three dogs receiving telmisartan had the dosage decreased. Twenty‐five dogs were receiving relatively low dosages (enalapril <0.8 mg/kg/day, benazepril <0.4 mg/kg/day, telmisartan <0.75 mg/kg/day) of RASi at ≥2 study visits. Eleven of these dogs received only telmisartan, 11 dogs received only an ACEi, and 3 transitioned from an ACEi to telmisartan.

**TABLE 1:** Dosages (median [IQR]) of the angiotensin converting enzyme inhibitors (ACEi) enalapril (E) and benazepril (B) and the angiotensin receptor blocker telmisartan (T) in 56 dogs

RASi treatment	Number of dogs	Dosage (mg/kg/d)	Final dosage (mg/kg/d)
No dose change—E	13	0.9 (0.6‐1.2)	n/a
No dose change—B	2	0.3 (0.2‐0.4)	n/a
No dose change—T	8	0.4 (0.4‐0.8)	n/a
Dose increase—E	9	0.5 (0.4‐0.8)	1.0 (0.6‐1.3)
Dose increase—B	4	0.4 (0.3‐0.8)	1.3 (0.5‐2.0)
Dose increase—T	5	0.5 (0.3‐0.7)	0.8 (0.5‐1.8)
Dose decrease—T	3	1.1 (0.8‐1.6)	0.6 (0.3‐1)

	Number of dogs	Dosage ACEi (mg/kg/d)	Dosage T (mg/kg/d)
Transition E to T	4	0.7 (0.3‐0.8)	0.5 (0.4‐0.9)
Transition B to T	5	0.5 (0.4‐0.7)	1 (0.7‐1.6)

	Number of dogs	Dosage ACEi (mg/kg/d)	Dosage T (mg/kg/d)
Both: E or B, plus T	3	1.46 (0.6‐2.0)	1.2 (0.7‐1.4)

*Note*: Twenty‐three dogs had no change in their monotherapy (no dose change), 18 dogs had a dose increase in their monotherapy (dose increase), 9 dogs transitioned from an ACEi to telmisartan (transition E/B to T), and 3 dogs received both an ACEi and telmisartan in combination on at least one study visit (E or B, plus T). Three dogs had a dosage decrease in telmisartan.

### 3.4 Urine aldosterone‐to‐creatinine ratio in proteinuric dogs

**FIGURE 1:**
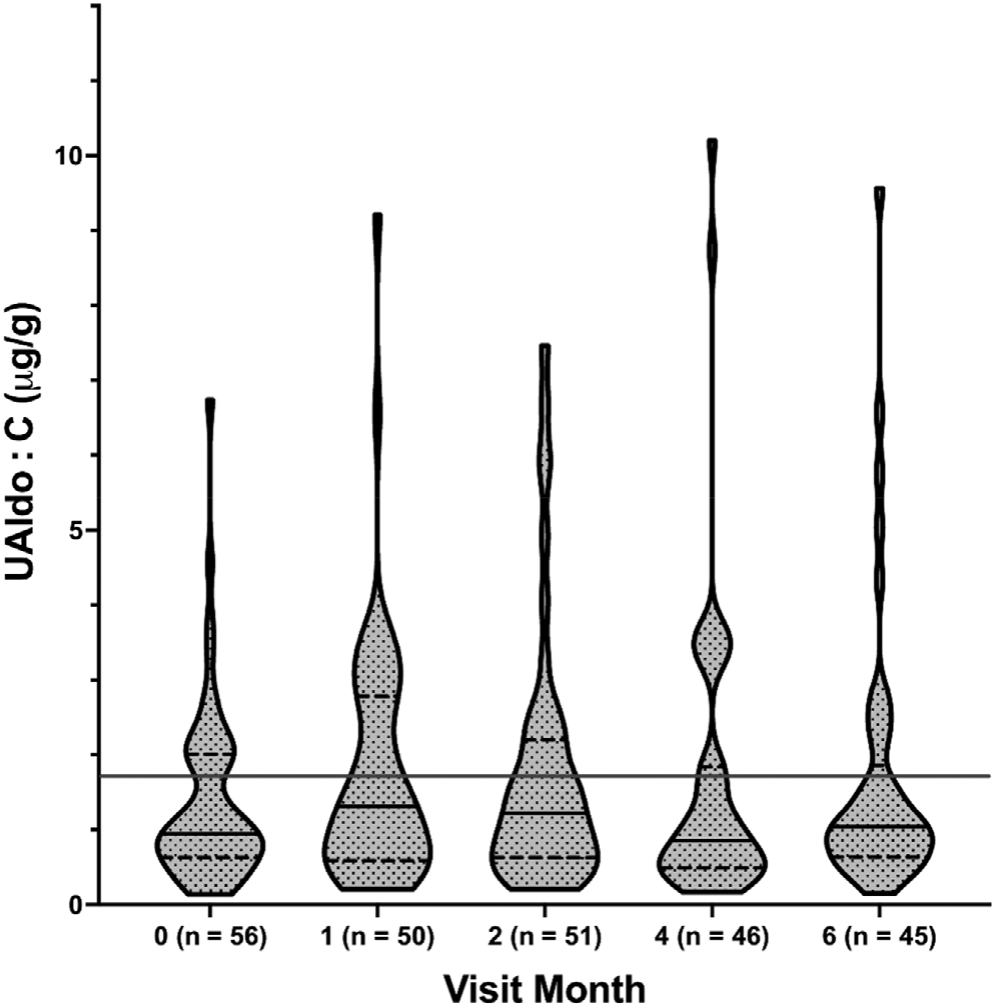
Median (solid line), interquartile range (dotted line), and distribution of the urine aldosterone to creatinine ratio (UAldo : C) for all 56 dogs. The number of dogs returning at each visit is noted. The long solid line indicates the 1.7 μg/g cutoff

The mean (SD) and median (IQR) UAldo : C ratios for each visit are shown in Table 2. The changes in this variable throughout the study are shown in Figure 1. The median (IQR) of the average UAldo : C for all study visits was 1.1 μg/g (0.6‐2.1). The median UAldo : C changed little throughout the study period and was below the 1.7 μg/g cutoff on all visits.

**TABLE 2:** Mean (SD) or median (interquartile range; IQR) of urine protein‐to‐creatinine ratio (UP : C), change in UP : C, urine aldosterone to creatinine ratio (UAldo : C), serum creatinine, albumin, potassium, and phosphorus concentrations, hematocrit (HCT), and blood pressure in 56 dogs with proteinuric chronic kidney disease

	Initial visit	Month 1	Month 2	Month 4	Month 6	
Variable	Median (IQR) or mean (SD), n = 56	Median (IQR) or mean (SD), n = 50	Median (IQR) or mean (SD), n = 51	Median (IQR) or mean (SD), n = 46	Median (IQR) or mean (SD), n = 45	Reference interval
UP : C	4.0 (2.6‐5.8)	3.2 (1.5‐5.2)	2.6 (1.4‐4.8)	2.6 (1.2‐5.8)	2.4 (1.3‐5.2)	< 0.5
UP : C change Visit 0‐6	–	–	–	–	0.9 (−0.7 to 2.8)	–
UAldo : C (μg/g)	1.0 (0.6‐2.0)	1.3 (0.6‐2.8)	1.2 (0.6‐2.2)	0.9 (0.5‐1.8)	1.0 (0.6‐1.9)	–
Creatinine (mg/dL)	1.3 (1.0‐2.1)	1.2 (1.0‐1.7)	1.3 (1.0‐2.1)	1.4 (0.9‐2.7)	1.4 (1.0‐2.8)	0.6‐1.6
Albumin (g/dL)	3.2 (2.8‐3.4)	3.2 (3.0‐3.5)	3.1 (2.9‐3.4)	3.1 (2.9‐3.5)	3.3 (2.9‐3.6)	3.0‐4.3
Potassium (mEq/L)	5.0 (0.49)	5.1 (0.44)	5.1 (0.54)	5.1 (0.55)	5.3 (0.58)	3.9‐5.4
Phosphorus (mg/dL)	4.2 (3.7‐5.0)	4.0 (3.3‐4.5)	3.8 (3.2‐4.8)	4.0 (3.4‐5.3)	4.4 (3.6‐5.3)	2.5‐6.0
HCT (%)	44 (7.3)	44 (7.4)	44 (6.5)	44 (6.7)	42 (8.7)	41‐58
Blood pressure (mm Hg)	154 (26)	157 (26)	152 (27)	156 (26)	152 (29)	<160

### 3.5 Aldosterone breakthrough

The cut off definition was applied to all dogs. Thirty‐four percent (19/56) of dogs had a UAldo : C that exceeded the 1.7 μg/g cutoff at ≥50% of visits during which they were receiving a RASi. Of these 19 dogs, 10 were receiving an ACEi, 5 were receiving telmisartan, and 4 were receiving both an ACEi and telmisartan, either having transitioned from ACEi to telmisartan or having been given an ACEi and telmisartan concurrently (Table 3).

**TABLE 3:** Summary of successful proteinuria reduction and occurrence of aldosterone breakthrough in the entire cohort (all RASi) and broken down by type of RASi and whether they were naïve to RASi at baseline or not

	Dogs with successful proteinuria reduction (%)	Dogs with ABT (cutoff definition) (%)	Dogs with ABT (baseline definition) (%)
All RASi, n = 56	20/56 (36)	19/56 (34)	–
ACEi only, n = 28	9 (32)	10 (36)	9 (32)
Telmisartan only, n = 16	6 (38)	5 (31)	3 (19)
Transition from ACEi to T or BOTH, n = 12	5 (42)	4 (33)	1 (8)
Dogs naïve to RASi at baseline, n = 22	8 (36)	–	13 (59)
Dogs receiving RASi at baseline, n = 34	12 (35)	–	–

Abbreviations: ABT, aldosterone breakthrough; ACEi, angiotensin converting enzyme inhibitor; BOTH, dogs received both an ACEi and telmisartan; RASi, renin‐angiotensin system inhibitor; T, telmisartan.

Twenty‐two dogs were naïve to RASi at their initial visit. Fifty‐nine percent (13/22) of these dogs had a UAldo : C ratios that exceeded their baseline (pre‐RASi) ratios at ≥50% of subsequent visits and met the baseline definition of ABT. Of these 13 dogs, 9 received an ACEi throughout the study, 3 received telmisartan throughout the study, and 1 dog received both an ACEi and telmisartan.

Eighteen dogs were receiving amlodipine at ≥1 visit. Nine dogs (50%) fit at least 1 definition of ABT, yet in only 4 dogs was the development of ABT possibly associated with amlodipine (ABT occurred after initiation of amlodipine or, for dogs receiving amlodipine through the study, ABT was present at every visit). Of the 38 dogs that were not receiving amlodipine, 12 (32%) fit at least 1 definition of ABT.

### 3.6 Proteinuria

**FIGURE 2:**
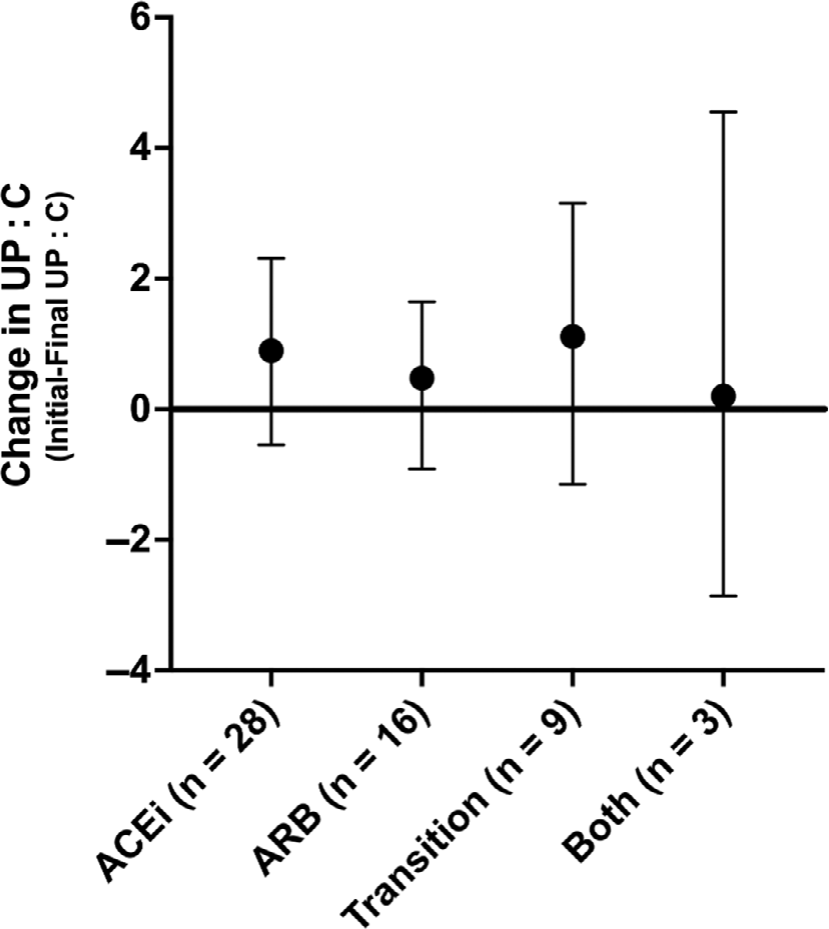
Median (dot) and interquartile range (bars) of the absolute value of change (initial visit‐final visit) in urine protein‐to‐creatinine ratio (UP : C) between baseline and the month 6 study visit. A negative value indicates that the UP : C increased. Dogs are grouped by the type(s) of renin‐angiotensin system inhibitor received. ACEi, angiotensin converting enzyme inhibitor only; ARB, angiotensin receptor blocker only; both, ACEi and ARB given concurrently on at least 1 study visit; transition, initiation of an ACEi, then transition to an ARB

The initial UP : C (mean [SD]) for specific treatments was: ACEi: 4.3 (2.0) and telmisartan: 3.5 (2.4). The median (IQR) for dogs on ACEi ultimately transitioned to telmisartan was 4.3 (3.0‐9.2) and for dogs receiving both an ACEi and telmisartan was 5.7 (4.0‐7.4). These UP : C ratios did not differ between groups (*P* = .13). The overall change (median [IQR]) in UP : C between visits (initial visit‐final visit) was ACEi: 0.9 (−0.5 to 2.3), telmisartan: 0.5 (−0.9 to 1.6), ACEi transition to telmisartan: 1.1 (−1.2 to 3.2), and ACEi and telmisartan: 0.2 (−2.9 to 4.6). The change in UP : C did not differ among these different treatment groups (*P* = .96; Figure 2).

**FIGURE 3:**
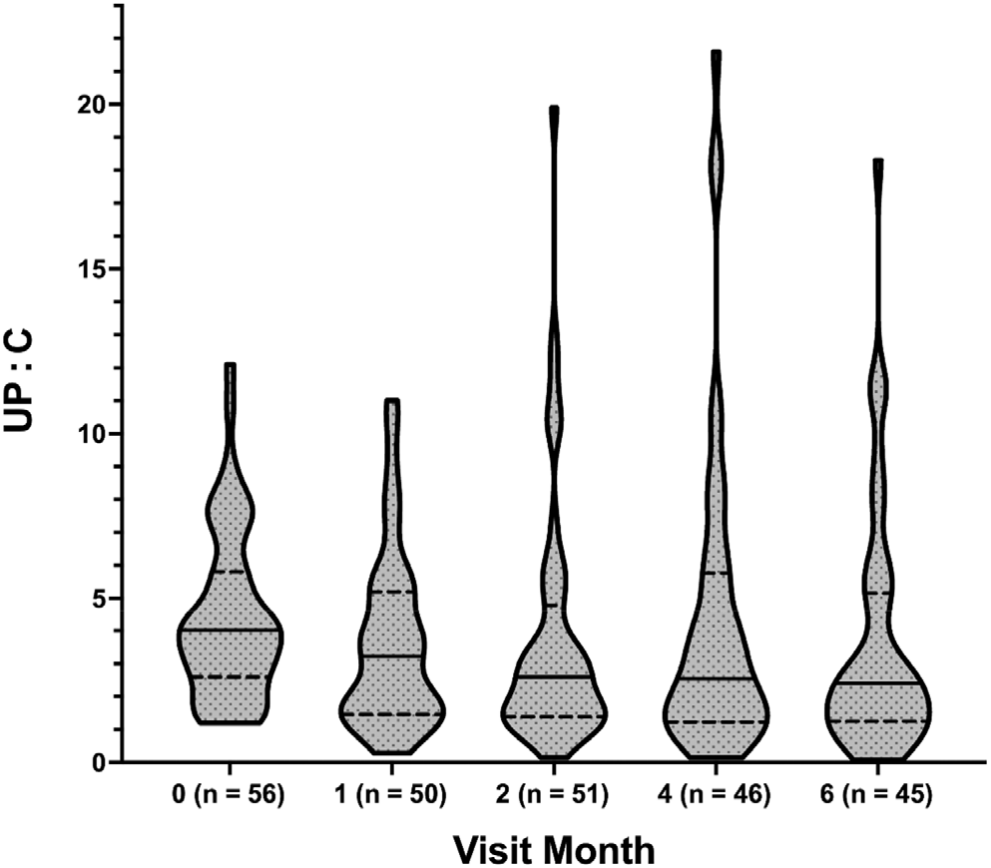
Median (solid line), interquartile range (dotted line), and distribution of urine protein‐to‐creatinine ratio (UP : C) for all 56 dogs. The number of dogs returning at each visit is noted

Thirty‐six percent (20/56) of dogs had a ≥ 50% decrease from their baseline UP : C on at least 50% of subsequent visits (Figure 3 and Table 3). Using this same definition, the overall success in UP : C decrease in dogs naïve to RASi was 8 of 22 dogs (36%) and in dogs already on a RASi was 12 of 34 dogs (35%). Of the 20 dogs with successful proteinuria reduction, 9 received only an ACEi, 6 received only telmisartan, and 5 transitioned from an ACEi to telmisartan or received both concurrently (Table 3).

### 3.7 Systemic hypertension

Eight dogs had poorly controlled systemic hypertension, defined as a SBP measurement ≥180 mm Hg at ≥2 visits (Table 2).

### 3.8 Variables influencing proteinuria reduction and aldosterone breakthrough

Two models evaluated the impact of variables on proteinuria reduction. In the first model, ABT was included as a variable using the cutoff definition, which was applied to all dogs. In the second model, ABT was included as a variable using the baseline definition, which was only applied to dogs not receiving a RASi at baseline. For the first model (cutoff definition), variables that passed the Akaike selection process included ABT (cutoff definition, yes/no), serum creatinine concentration (>2.8 mg/dL, yes/no), and time. The only variable significantly associated with successful proteinuria reduction was time. Longer duration in the study was associated with higher likelihood of successful proteinuria reduction (*P* = .002; odds ratio, 1.6; 95% CI, 1.2‐2.2). For the second model, variables that passed the Akaike selection process included ABT (baseline definition, yes/no), serum creatinine concentration (>2.8 mg/dL, yes/no), and RASi dosage escalation (yes/no). None of these variables was significantly associated with successful proteinuria reduction.

A third model evaluated the influence of variables on the occurrence of ABT (cutoff definition), which allowed the inclusion of all dogs. Variables that passed the Akaike selection process included serum creatinine concentration (>2.8 mg/dL, yes/no), RASi dosage escalation (yes/no), and time. Dogs with a serum creatinine concentration >2.8 mg/dL had a lower likelihood of ABT (*P* = .3; odds ratio, 0.09; 95% CI, 0.01‐0.8). No other variable was significantly associated with ABT.

No association was found between ABT (by any definition at ≥50% of visits) and successful proteinuria reduction (*P* = .59). Evaluating ABT (yes/no) and UP : C decrease from baseline (yes/no) on all visit days also did not show an association (baseline definition of ABT, *P* = .23; cutoff definition of ABT, *P* = .58). Negligible but positive correlation was found between UAldo : C and UP : C (r = 0.09; *P* = .12).

## 4 DISCUSSION

Depending on the definition used, ABT occurred in 34% to 59% of dogs receiving RASi for the treatment of CKD_P_. Thirty‐six percent of dogs receiving an ACEi, telmisartan, or both, had durable proteinuria reduction during the study period. Serum creatinine concentration, ABT, and RASi dosage escalation were not associated with proteinuria reduction. Only time (longer duration in the study) was associated with a higher likelihood of successful proteinuria reduction.

Failure of RASi to suppress aldosterone (i.e., ABT) was not associated with proteinuria outcome. This finding was surprising because excess aldosterone damages the final filtration barrier in the glomerulus, specifically the podocyte.^10^ This result could indicate that, on average, aldosterone concentrations were not sufficiently increased or not increased for a long enough time to cause progressive glomerular damage or that aldosterone excess was not a major mediator of proteinuria in this population. Whether the addition of a MRA, such as spironolactone, to the treatment of proteinuria in dogs with CKD_P_ will provide additional benefit remains to be determined. Theoretical benefits of MRA include decreased inflammation and fibrosis.^23^ This class of drug improves proteinuria reduction in people with CKD_P_ and is used in the setting of persistent proteinuria despite use of an ACEi or ARB or both.^24^ Ultimately, the only variable significantly associated with successful proteinuria reduction in our study was time. This finding may reflect improved drug efficacy with longer duration of treatment or that dogs with higher treatment success were more likely to return for subsequent visits. Dosage escalation was not found to be associated with successful proteinuria reduction. Because dosage escalation was at the clinician's discretion, dosage escalation may have been more common in cases where proteinuria was resistant to the initial type of treatment.

Sixty‐one percent of dogs (34/56) in our study were already receiving a RASi at the initial examination. Most of these dogs had been on a RASi for <2 months. The success in proteinuria reduction (≥50% decrease from baseline at ≥50% of subsequent visits) was similar between dogs naïve to RASi (8/22; 36%) and dogs already on a RASi (12/34; 35%). Angiotensin converting enzyme inhibitors and telmisartan performed similarly, with 32% and 38% of dogs receiving either an ACEi or telmisartan, respectively, having a durable proteinuria reduction. The comparison of efficacy between these RASi must be interpreted taking into consideration the uncontrolled and nonrandomized design of our study. The dosages and types of RASi used were at the discretion of the attending clinicians, who were board‐certified small animal internal medicine diplomates or residents under diplomate supervision. A limitation to this approach is that the treatment groups were not balanced in numbers and may have had relevant baseline differences. Additionally, the selection of RASi type and dosage was not randomized and may have been biased by several factors. An advantage of the approach however is that it reflects actual specialty practice. Despite this patient‐centered approach and frequent follow‐up, proteinuria reduction was modest, with 64% of dogs not achieving proteinuria reduction from baseline at ≥50% of visits and 20 dogs (36%) experiencing an increase in UP : C between their first and last visit. As can be seen in Table 1, the median RASi dosages were relatively low in comparison to a dosage escalation protocol utilized previously.^8^ This difference may explain the lower proteinuria reduction success rate in our study. Additionally, in 8 dogs, SBP was not adequately controlled. Recent studies have investigated the ideal RAAS inhibition strategy in CKD_P_. The ARB telmisartan has been shown to result in a higher percentage decrease in proteinuria than the ACEi enalapril in dogs with CKD_P_ during short‐term treatment.^8^ Previous studies in people have shown that dual treatment with the combination of ACEi and ARB leads to a higher proteinuria reduction when compared to either agent alone.^25^ A recent retrospective study in dogs also supports this finding.^26^ Recent meta‐analyses in people, however, have found a higher risk for hyperkalemia, acute kidney injury, and death in patients receiving combination treatment.^27^, ^28^

When our study was designed, comprehensive evaluation of RAAS was not readily available. The recent development of RAAS‐specific high‐performance mass spectrometry assays has changed this limitation. Future studies evaluating the efficacy of RAAS inhibitor treatment and impact of this hormone system on outcomes will benefit from comprehensive profiling of this system. The UAldo : C ratio has some advantages. The urine sample can be collected at home, it quantifies the terminal hormone of the renin‐angiotensin‐aldosterone system, has been correlated with 24‐hour urine aldosterone secretion,^16^ and likely reflects a dog's overall RAAS activity. The Bland‐Altman analysis of UAldo : C in the normal dogs suggests that it could be expected that a dog with a UAldo : C on day 1 of 1.0 μg/kg could have a UAldo : C between 0.44 and 1.57 μg/g on day 2. This variation may arise from several factors including circadian variations in aldosterone secretion,^29^ hydration status,^30^ dietary sodium intake,^31^ and stress. Intra‐individual day‐to‐day variation in the USG has been documented in healthy dogs,^32^ and it is likely that RAAS activity fluctuates as fluid and electrolyte balance and blood pressure vary in normal dogs. This variation is likely the main contributor to inter‐dog variation, but breed, sex, and age^33^ also may contribute. Urinary aldosterone concentrations also may fluctuate over longer periods of time because of variations in local podocyte production, hepatic metabolism, and increased concentrations of other secretagogues (adrenocorticotropic hormone, thyroid hormone). Given the negligible correlation between UP : C and UAldo : C, it is unlikely that glomerular permselectivity is a major determinant of urinary aldosterone secretion. Although moderate day‐to‐day variation limits the use of UAldo : C as a point of care biomarker, use of an ABT definition that required multiple timepoints likely decreased the impact of this variation. In people, blood aldosterone concentrations have been shown to be inversely associated with estimated glomerular filtration rate.^34^ The finding that a higher likelihood of ABT was associated with lower serum creatinine concentration is therefore difficult to explain.

The upper, and more clinically relevant, limit of the UAldo : C reference interval derived from the normal dogs was imprecise with a WCI/WRI ratio of 0.77. This finding is likely a consequence of individual variation and small sample size, which is unlikely to result in a WCI/WRI less than the desirable 0.2.^35^ Although reference interval determination in veterinary species should include at least 120 subjects,^36^ this number is likely higher for UAldo : C given the WCI/WRI of these 31 dogs. Because of this imprecision, we generated a cutoff for the ABT definition by calculating the mean plus 2 SD for this cohort of healthy dogs, which was 1.7 μg/g.

Our study had strengths and limitations. One strength was its longitudinal study design that allowed for evaluation of outcomes over a 6‐month period, which is longer than many published proteinuria studies. We assessed the prevalence of ABT in a population of dogs being treated for proteinuria in actual clinical practice, which is both a strength and limitation. The primary limitation of this approach is the heterogeneity of the RASi treatment strategy. Dosages of RASi were sometimes lower than suggested, which probably reflects clinician concerns about causing or aggravating azotemia. Findings comparing RASi treatment efficacy must be interpreted considering the nonrandomized, uncontrolled nature of our study. Diet and diet changes also were not controlled and the impact of this design feature on ABT could not be determined. Ideally, hypertension would have been controlled before the initiation of the study. The use of the vasodilator amlodipine in 18 dogs also may have increased the prevalence of ABT, but in only 4 dogs was the administration of amlodipine possibly associated with the development of ABT. Additionally, as is typical in clinical practice, the underlying etiology of the proteinuria was not definitively diagnosed. Consequently, the etiologies of proteinuria in these patients likely varied. A uniform and comprehensive systemic screening for occult infectious or systemic diseases was not carried out in all dogs, and some dogs may have had an occult infectious disease process contributing to their proteinuria. Trace proteinuria was common in the 31 healthy dogs. Although dogs were excluded if they had more than trace proteinuria or trace proteinuria with USG <1.030, it is possible that some of these dogs had clinically relevant proteinuria. Finally, the UAldo : C ratio has moderate day‐to‐day variability and is not an ideal point‐of‐care biomarker. Longitudinal monitoring of this biomarker however likely provides a general assessment of a patient's RAAS activity.

In conclusion, ABT was common in dogs receiving RAS inhibitors for CKD_P_, but it did not appear to influence the success of proteinuria reduction. In this cohort, the use of ACEi or telmisartan at the dosages evaluated was associated with modest proteinuria reduction during the 6‐month study period. The UAldo : C ratio has moderate day‐to‐day variation in normal dogs and is likely best assessed longitudinally.

## CONFLICT OF INTEREST DECLARATION

Dr Ames has received consulting fees, research support, and honoraria from Ceva Sante Animale; Dr Vaden has received honoraria from Boehringer‐Ingelheim; Dr Atkins has received consulting fees, research support, and honoraria from Boehringer‐Ingelheim, Ceva Sante Animale, and Vetoquinol; Jean‐Sebastien Palerme has received consulting fees from Antech Diagnostics, Infiniti Medical; Catherine E. Langston has received consulting fees from Elanco; Gregory F. Grauer has received consulting fees, research support, and honoraria from Boehringer‐Ingelheim, IDEXX, and Vetoquinol. Dr Vaden serves as Associate Editor for the Journal of Veterinary Internal Medicine. She was not involved in review of this manuscript. Sarah Shropshire, Christina Bove and Tracy Webb have no disclosures.

## INSTITUTIONAL ANIMAL CARE AND USE COMMITTEE (IACUC) OR OTHER APPROVAL DECLARATION

Approvals from IACUC and Clinical Review Boards were obtained at each institution.

## HUMAN ETHICS APPROVAL DECLARATION

Authors declare human ethics approval was not needed for this study.

## Supporting information


**Figure S1**. Median (solid line), interquartile range (dotted line), and distribution of urine aldosterone to creatinine ratio (UAldo : C) for 31 healthy dogs. The mixed sample contained equal aliquots of day 1 and 2 urine.

**Table S1**. Pertinent urinalysis and serum biochemistry parameters and PCV from 31 healthy dogs.
**Table S2**. Urine aldosterone, urine creatinine, and reference intervals and 90% confidence intervals for the lower and upper limits and the WCI/WRI for the urine aldosterone to creatinine (UAldo : C) reference interval (n = 31 healthy dogs). WCI/WRI: width of the 90% confidence interval of the limits to width of the reference interval.

**Appendix S1** Supporting Information
