# Supplementary material for: Prevalence of aldosterone breakthrough in dogs receiving renin‐angiotensin system inhibitors for proteinuric chronic kidney disease
Source: J Vet Intern Med. 2022 Nov 9;36(6):2088–97. doi: 10.1111/jvim.16573 (PMC9708418; doi:10.1111/jvim.16573)

Supplemental Figure 1. Median (solid line), interquartile range (dotted line), and distribution of urine aldosterone to creatinine ratio (UAldo:C) for 31 healthy dogs. The mixed sample contained equal aliquots of day 1 and 2 urine.

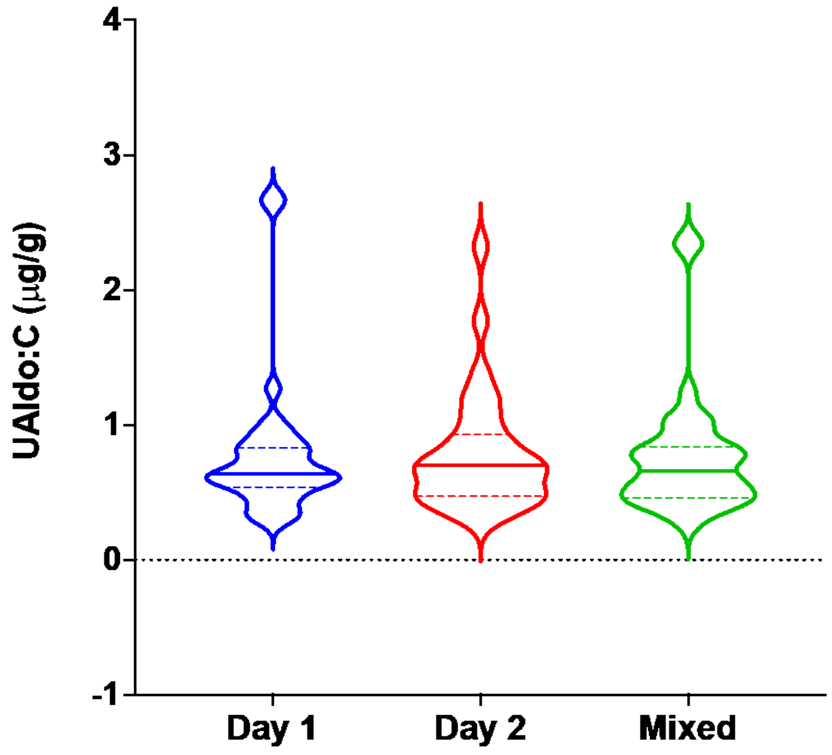

Supplement: Supplementary file 1 — Figure S1. Median (solid line), interquartile range (dotted line), and distribution of urine aldosterone to creatinine ratio (UAldo : C) for 31 healthy dogs. The mixed sample contained equal aliquots of day 1 and 2 urine. [file JVIM-36-2088-s001.pdf]
