# Supplementary material for: Prevalence of aldosterone breakthrough in dogs receiving renin‐angiotensin system inhibitors for proteinuric chronic kidney disease
Source: J Vet Intern Med. 2022 Nov 9;36(6):2088–97. doi: 10.1111/jvim.16573 (PMC9708418; doi:10.1111/jvim.16573)
Supplement: Supplementary file 2 — Table S1. Pertinent urinalysis and serum biochemistry parameters and PCV from 31 healthy dogs. Table S2. Urine aldosterone, urine creatinine, and reference intervals and 90% confidence intervals for the lower and upper limits and the WCI/WRI for the urine aldosterone to creatinine (UAldo : C) reference interval (n = 31 healthy dogs). WCI/WRI: width of the 90% confidence interval of the limits to width of the reference interval. [file JVIM-36-2088-s003.pdf]

Supplemental Table 1: Pertinent urinalysis and serum biochemistry parameters and PCV from 31 healthy dogs.

Parameter	Median (IQR)	Mean (SD)	Range	Reference Interval
**USG**	1.043 (1.035 - 1.053)	1.043 (0.010)	1.017 - 1.064	1.015-1.045
**BUN (mg/dL)**	16 (14 - 19)	16.84 (3.82)	10 - 29	7 - 30
**Creatinine (mg/dL)**	1.2 (1.0 - 1.3)	1.13 (0.18)	0.7 - 1.4	0.6 - 1.6
**Total Protein (g/dL)**	6.3 (6.1 - 6.5)	6.31 (0.30)	5.8 - 7.3	5.0 - 7.0
**Albumin (g/dL)**	3.7 (3.6 - 3.9)	3.72 (0.27)	2.7 - 4.1	3.0 - 4.3
**Na^+ (mEq/L)**	149 (147 - 150)	148.7 (2.21)	144 - 154	142 - 152
**K^+ (mEq/L)**	4.53 (4.30 - 4.73)	4.51 (0.32)	3.69 - 5.24	3.9 - 5.4
**Cl^- (mEq/L)**	111.8 (110.4 - 114.0)	112.1 (2.24)	108.8 - 117.7	108 - 118
**PCV (%)**	51 (48 - 56)	51.97 (5.44)	42 - 64	35 - 57

Supplemental Table 2: Urine aldosterone, urine creatinine, and reference intervals and 90% confidence intervals for the lower and upper limits and the WCI/WRI for the urine aldosterone to creatinine (UAldo:C) reference interval (n=31 healthy dogs). WCI/WRI: width of the 90% confidence interval of the limits to width of the reference interval.

Parameter	Median (IQR)	Mean (SD)	Range	Reference Interval	90% CI of Limits	WCI/WRI
**Urine Aldosterone (pmol/L) Day 1**	5235 (3195 - 7973)	5591 (2812)	1 - 13290	n/a	n/a	n/a
**Urine Aldosterone (pmol/L) Day 2**	5370 (3690 - 7620)	6021 (3032)	2 - 13590	n/a	n/a	n/a
**Urine Aldosterone (pmol/L) Mixed**	5670 (3690 - 7650)	5900 (2480)	1530 - 12600	n/a	n/a	n/a
**Urine Creatinine (mmol/L) Day 1**	24 (17 - 35)	26 (10)	9.7 - 48	n/a	n/a	n/a
**Urine Creatinine (mmol/L) Day 2**	22 (20 - 37)	28 (11)	8.3 - 52	n/a	n/a	n/a
**Urine Creatinine (mmol/L) Mixed**	24 (19 - 34)	27 (9.8)	9.0 - 49	n/a	n/a	n/a
**UAldo:C (µg/g) Day 1**	0.64 (0.54 - 0.83)	0.79 (0.55)	0.28 - 2.7	0.23 - 1.82	Lower: 0.17-0.32<br>Upper: 1.27-2.5	0.09 and 0.77
**UAldo:C (µg/g) Day 2**	0.70 (0.47 - 0.93)	0.79 (0.44)	0.31 - 2.3	0.26 - 1.89	Lower: 0.20-0.32<br>Upper: 1.40-2.35	0.07 and 0.58
**UAldo:C (µg/g) Mixed**	0.66 (0.46 - 0.84)	0.78 (0.48)	0.27 - 2.4	0.24 - 1.81	Lower: 0.18-0.33<br>Upper: 1.28-2.50	0.10 and 0.78
