# Supplementary material for: Prevalence of aldosterone breakthrough in dogs receiving renin‐angiotensin system inhibitors for proteinuric chronic kidney disease
Source: J Vet Intern Med. 2022 Nov 9;36(6):2088–97. doi: 10.1111/jvim.16573 (PMC9708418; doi:10.1111/jvim.16573)
Supplement: Supplementary file 3 — Appendix S1 Supporting Information [file JVIM-36-2088-s002.pdf]

Supporting information

Healthy Dogs – Demographic and Clinical Results

Thirty-one dogs, 17 spayed females and 14 neutered males, were included. Their age (median; IQR) was 4.6 (3.6-6.6) years, and their body weight (median; IQR) was 22.2 (14.7-31.5) kilograms. Twenty-four dogs had urine sampled one day apart, whereas seven had urine sampled two days apart. Twenty-seven dogs were primarily eating a commercial dog food diet where the percent sodium content on a dry matter basis was published and ranged from 0.2 to 0.54%. All dogs were predominantly eating a dry dog food and receiving a variety of treats. There was no significant correlation between diet sodium content and day 1 UAldo:C. All dogs were adequately hydrated based on a physical examination.

Sixteen dogs had trace proteinuria; in all dogs the urine specific gravity (USG) was greater than 1.030; in 13 of these 16 dogs the USG was greater than 1.040 (Supplemental Table 1). One dog had a PCV of 64% and TS of 6.8g/dL. This dog was not overtly dehydrated on physical examination. Splenic contraction was the supposed mechanism for this finding, though further follow-up was not performed. No clinically relevant abnormalities were found on serum biochemistry in any dog: one dog had a mild elevation in ALP (151 IU/L; normal 15-140), and one dog had a mildly decreased albumin (2.7 g/dL; normal 3.0-4.3) concentration.
